# Taking time: Auditory statistical learning benefits from distributed exposure

**DOI:** 10.3758/s13423-024-02634-w

**Published:** 2025-01-17

**Authors:** Jasper de Waard, Jan Theeuwes, Louisa Bogaerts

**Affiliations:** 1 Department of Experimental and Applied Psychology, Vrije Universiteit Amsterdam, Van der Boechorststraat 7, 1081 BT Amsterdam, The Netherlands; 2 Institute Brain and Behavior Amsterdam (iBBA), Amsterdam, Netherlands; 3 William James Center for Research, ISPA–Instituto Universitario, Lisbon, Portugal; 4 Department of Experimental Psychology, Ghent University, Ghent, Belgium

**Keywords:** Statistical learning, Auditory, Spacing effect, Distributed learning, Language learning, Longitudinal

## Abstract

In an auditory statistical learning paradigm, listeners learn to partition a continuous stream of syllables by discovering the repeating syllable patterns that constitute the speech stream. Here, we ask whether auditory statistical learning benefits from spaced exposure compared with massed exposure. In a longitudinal online study on Prolific, we exposed 100 participants to the regularities in a spaced way (i.e., with exposure blocks spread out over 3 days) and another 100 in a massed way (i.e., with all exposure blocks lumped together on a single day). In the exposure phase, participants listened to streams composed of pairs while responding to a target syllable. The spaced and massed groups exhibited equal learning during exposure, as indicated by a comparable response-time advantage for predictable target syllables. However, in terms of resulting long-term knowledge, we observed a benefit from spaced exposure. Following a 2-week delay period, we tested participants’ knowledge of the pairs in a forced-choice test. While both groups performed above chance, the spaced group had higher accuracy. Our findings speak to the importance of the timing of exposure to structured input and also for statistical learning outside of the laboratory (e.g., in language development), and imply that current investigations of auditory statistical learning likely underestimate human statistical learning abilities.

**Supplementary Information:**

The online version contains supplementary material available at 10.3758/s13423-024-02634-w.

## Introduction

Since the seminal work by Ebbinghaus (1885), on human learning and forgetting, evidence for superior learning outcomes achieved through several spaced practice sessions compared with intensive massed learning has continued to grow (review: Gerbier & Toppino, 2015). Conventionally, the spacing effect is studied using learning paradigms in which participants study materials such as word lists (e.g., Melton, 1967), texts (e.g., Rawson & Kintsch, 2005), or faces (e.g.,Russo et al., 1998) and are tested on these materials after a delay period (Wiseheart et al., 2019). The testing phase assesses the ability to recall or recognize items that were learned either in one massed session or across several spaced sessions that total the same length. However, learning in daily life often takes place incidentally, especially concerning the extraction of statistical regularities (e.g., Aslin, 2017; Saffran et al., 1996). For this so-called statistical learning, where learners extract stimuli from a continuous input stream rather than being presented with clearly separated individual stimuli, the spacing effect could also exert its influence.

Statistical learning is the ability to extract statistical patterns from the environment and use them to segment sensory input, ultimately allowing the anticipation of upcoming events (Theeuwes et al., 2022). A seminal study by Saffran et al. (1996) exposed 8-month-old infants to a continuous stream of spoken syllables. They demonstrated that infants could learn the underlying structure of the speech stream from passive listening, and could use this information to parse the stream into its constituents (syllables consistently occurring together making up novel words). Since then, research in statistical learning has expanded to include learning in adults (Saffran et al., 1997), primates (Conway & Christiansen, 2001; Hauser et al., 2001), and rats (Toro & Trobalón, 2005), as well as over different auditory and visual input materials (Frost et al., 2019). Statistical learning paradigms range from passive perception to active engagement (Arciuli & Simpson, 2012; Batterink 2017), with the latter usually involving some kind of cover task, such as detecting a direct repetition of a stimulus (e.g., Arciuli & Simpson, 2012; Emberson et al., 2011; Turk-Browne et al., 2005). The current consensus is that statistical learning does not require conscious effort and that learning can occur without awareness (Frost et al., 2019), although the absence of awareness is often hard to establish (Vadillo et al., 2016, 2020) and the role of attention remains a contended issue (Batterink & Paller, 2019; Duncan & Theeuwes, 2020; Toro et al., 2005; Turk-Browne et al., 2005). However, evidence suggests that the explicitness of the task and awareness of the participants have little consequences for the eventual learning (Batterink et al., 2015; Gao & Theeuwes, 2022; see also Ordin & Polyanskaya, 2021).

Considering the domain of language, statistical learning plays a key role in the rapid language acquisition of infants and children (e.g., Abreu et al., 2023; Erickson & Thiessen, 2015; Romberg & Saffran, 2010), and individuals’ statistical ability is predictive of individual differences in oral language and literacy skills (Ren et al., 2023; Siegelman et al., 2017). The spacing effect has been observed in many language-learning experiments (Bahrick et al., 1993; Bird, 2011; Rogers 2015, 2017), yet results have been equivocal. For example, Pagán and Nation (2019) did not find a spacing effect for adults learning new words from sentence context, and a recent study demonstrated improved recognition of written word forms but comparable recall after spaced learning (Wegener et al., 2022). Considering the importance of statistical learning for language acquisition, it is remarkable that it has never been studied in relation to statistical learning. Indeed, nearly all studies within the field of statistical learning have employed a single-session exposure phase, and most work tested immediate recognition of the embedded patterns (Frost et al., 2019). Yet recent findings suggest that even single-session exposure leads to stable memory representations of the learned patterns, at least up to 1 week later (Arciuli & Simpson, 2012). This raises the question of the optimal exposure regimen for this incidental and more automatic type of learning. Specifically, we asked whether auditory statistical learning would benefit from a spaced exposure phase.

## Predictions

Based on theoretical views stressing the competition between different memory systems, such as the complementary learning systems theory (McClelland et al., 1995) and the proposal of competing declarative and procedural learning systems (Foerde et al., 2006), one might predict that statistical learning performance does not necessarily mimic the advantage of distributed learning found for intentional learning tasks. While episodic memory encodes individual events, statistical learning relies on the recurring relations between events to extract regularities, which has led to the idea of a neurocognitive trade-off between episodic memory and statistical learning (Schapiro et al., 2017; Sherman & Turk-Browne, 2020; Sherman et al. 2022). Similarly, recent findings suggest that cognitive depletion may improve the capacity for statistical learning (Smalle et al., 2022) and language learning (Smalle et al., 2021), allowing it to occur uninhibited by higher cognitive mechanisms. This suggests that explicit learning strategies and statistical learning may rely on competing neural mechanisms. The absence of a spacing benefit for auditory statistical learning would thus speak to the dissociation between statistical learning and more conventional learning paradigms.

Other authors, however, have argued that statistical learning is not as unique as it purports to be, and relies on the same neural mechanisms that govern any memory process (Perruchet & Vinter, 1998; Thiessen, 2017). Furthermore, the spacing effect could reflect a fundamental characteristic of human memory independent of the specific type of learning. Indeed, suggestive of the generality of the spacing effect is that it has been observed in infants (Cornell, 1980) and first-graders (Toppino & DiGeorge, 1984). Using conditioning and habituation paradigms, distributed learning has also been found to benefit even organisms with minimal cognitive abilities, such as fruit flies (Tully et al., 1994), sea slugs (Carew et al., 1972), and bees (Deisig et al., 2007). Given the widespread advantages of spaced learning, we preregistered the prediction that auditory statistical learning in adults would likewise benefit from a spaced exposure phase.

## Current study

**Fig. 1:**
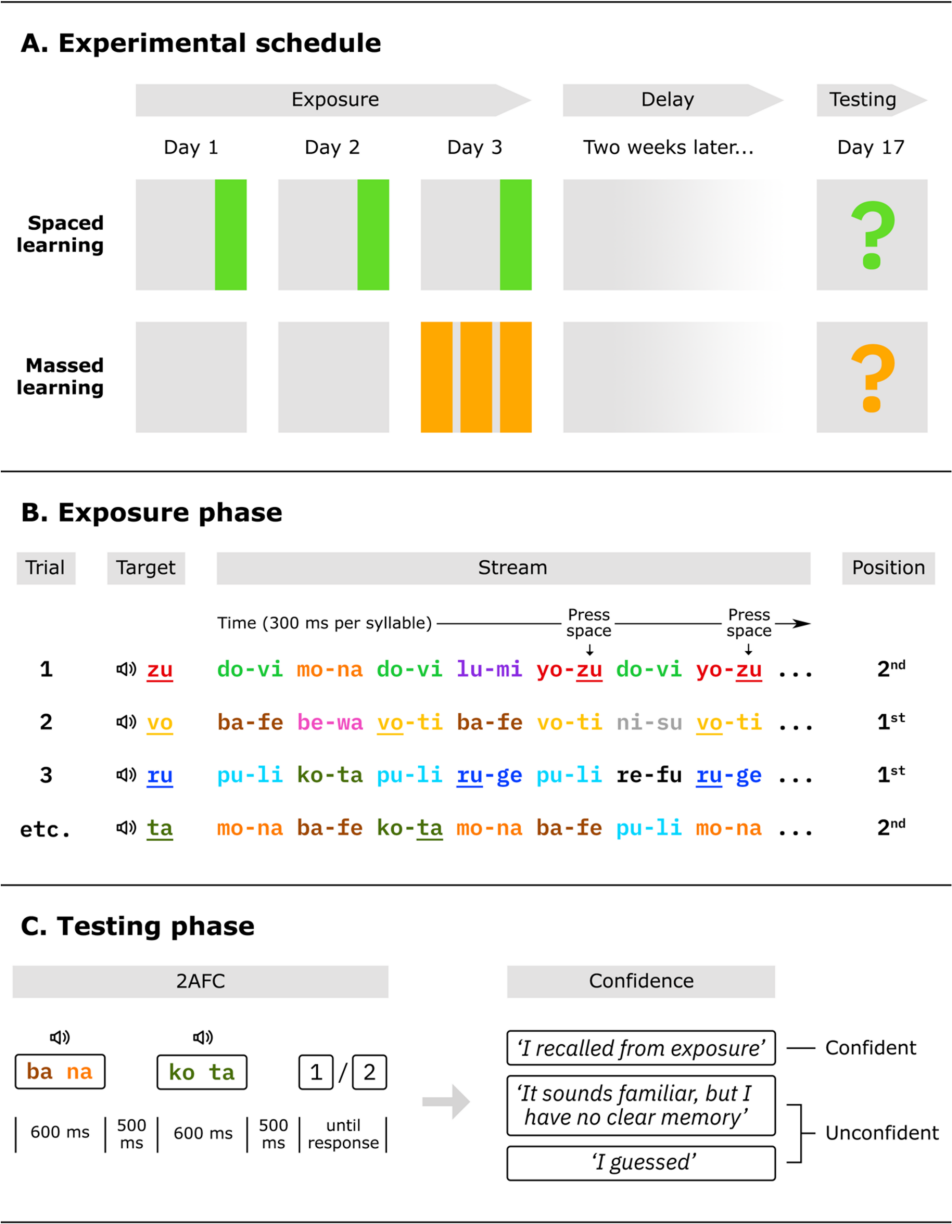
Overview of the experimental design (we recommend viewing in color). **A.** The spaced group’s exposure phase was spread out over 3 days, while the massed group’s took place in 1 day. After a 2-week delay, both groups performed the testing phase. **B.** In the exposure phase, participants responded (timed) to a prespecified target (e.g., “zu”) in a rapid auditory stream of syllables. The stream was composed of fixed syllable pairs (e.g., “yo zu”) that remained constant throughout the experiment. **C.** In the testing phase, participants chose one of two auditory pairs (one from the learning phase, one a newly created “foil”) as most familiar, and subsequently indicated their confidence for their choice. (Color figure online)

We used a longitudinal design with two groups (Fig. 1A). The spaced group was exposed to the auditory regularities in three single block sessions spread out over 3 consecutive days, while in the massed group all exposure occurred in a single session of three blocks on the third day. In the exposure phase (Fig. 1B), participants listened to rapid streams of syllables while responding as quickly as possible to a target syllable that was defined at the start of each stream. Streams were made up of syllable pairs that remained fixed throughout the entire experiment. Once learned, the first syllable of a pair can serve as a cue for the second syllable (e.g., Batterink, 2017), such that we expected faster response times (RTs) when the target syllable was second compared with first in the pair. The inclusion of the target-detection task required participants to actively listen, but since none of the instructions or feedback referred to the regularities in the streams, the learning can still be considered incidental. Since the spacing effect is most pronounced after a delay period (Cepeda et al., 2008), we incorporated a 2-week delay after the exposure phase. In the subsequent testing phase (Fig. 1C), participants performed a two-alternative forced-choice (2AFC) test, in which pairs from the exposure stream were pitted against foils, and they were required to indicate the more familiar pair followed by a confidence rating. We expected above-chance performance for confident as well as unconfident responses, with elevated performance in the spaced group. The study was preregistered online (https://aspredicted.org/e4mm3.pdf).

## Methods

### Participants

In the absence of a reliable effect size from previous literature, we followed Brysbaert (2019), who suggests *d* = 0.4 as the smallest theoretically meaningful effect size in psychological research. To achieve *d* = 0.4 with β = 0.80, we used a sample size of 200 for a between-groups comparison. We ran two iterations of the study to replace participants who dropped out, after which each group contained 99 participants. All participants took part in the study through Prolific (Palan & Schitter, 2018) and were between 18 and 40 years old (spaced group mean age = 31.1 years, massed group mean age = 31.5 years). They reported living in the UK or Ireland, and having at minimum an undergraduate degree. Participation took approximately 35 min, earning £5.25 plus a £2 bonus after completion of all sessions. The experiment was approved by the Ethical Committee of the Faculty of Behavioral and Movement Sciences of the Vrije Universiteit Amsterdam. All participants gave informed consent and all methods were performed in accordance with the Declaration of Helsinki.

### Experimental design

The experiment was created in OpenSesame (Mathôt et al., 2012) using OSweb 1.4.11, and run using JATOS 3.7.4 (Lange et al., 2015). We used the 24 male-voice auditory syllable stimuli from Batterink (2017). Each syllable had a duration of 300 ms. Audio was played at a comfortable volume determined by the participant at the start of each session. The display background was grey (RGB: 128/128/128).

Depending on their (randomly determined) group assignment, of which participants were not informed, they were asked to return to the experiment on Prolific at set dates, following the schedule in Fig. 1A. The exposure phase consisted of three blocks of 24 trials, and the testing phase was a single block of 36 trials. To investigate a possible link between statistical learning and cognitive depletion, participants indicated their wakefulness on a Likert scale (1–5) at the beginning and end of every block. For each participant, the 24 syllables were randomly arranged into 12 syllable pairs, which remained constant throughout the experiment.

In the exposure phase (Fig. 1B), every trial started with a target syllable, which could be repeated by pressing the “a” key. The stream started after a 500-ms delay once the participant pressed the “s” key. Each syllable had a duration of 300 ms with 0 ms between syllables, and each stream contained the target four times. Audio from an example trial is available online (https://osf.io/5smzf/). Participants were instructed to press the space bar as soon as they heard the target. A response was considered correct if it fell within a 100–1,200-ms time window after target onset (see the Supplementary Materials for a wider time window comparison). Feedback was provided after every stream, indicating the number of correct responses (out of four) and average RT. If a participant responded incorrectly more than twice during a stream (indicating that they could be blindly pressing the space bar), a warning message was shown.

Each stream contained four unique syllable pairs, repeated four times for a total of 32 syllables. The pairs were shuffled pseudorandomly so that (i) a pair is never directly repeated (AA), (ii) the same combination of pairs is never directly repeated (ABAB), and (iii) the target is not in the first or last pair of the stream. Within a block of 24 streams, each syllable served as the target once, and each set of four consecutive streams contained two targets that were first within their pair (e.g., *ko* ta), and two targets that were second within their pair (e.g., ko *ta*). Furthermore, each set of three consecutive streams contained all 24 syllables. These constraints were implemented to ensure gradual learning of all pairs, and to minimize measurement error as a consequence of being early or late in a block. After the last block of the exposure phase, participants were asked two questions to assess their awareness of the regularities: (Q1) “This experiment was made up of a collection of audio streams. Did you notice anything about the streams?” and (Q2) “The order of the sounds in the streams was not random. Each stream consisted of several repeating subgroups of sounds. Please estimate: how many sounds were in a subgroup?”

For the testing phase (Figure 1C), 12 foil pairs were created by recombining the syllables from the learned pairs, respecting each syllable’s position (i.e. if a syllable had the second position within the learned pair, it would also have the second position within the foil). Each test trial pitted a learned pair and a foil against each other. Each learned pair was pitted against three different foils, making 36 trials. The trial order was pseudorandom, such that in every 12 consecutive trials, all learned pairs and all foils were used once, half of the trials started with the foil, and the syllables from the learned pair in a given trial were never also in the foil.

When a participant pressed “s” to start the trial, a 1,000-ms delay was followed by the first pair/foil (300 ms per syllable), a 500-ms delay, the second pair/foil, another 500-ms delay, and a response display. Participants indicated the most familiar pair by pressing 1 or 2. After a response was provided, participants rated their confidence by selecting one of three options: (i) “I recalled from exposure”; (ii) “It sounds familiar, but I have no clear memory”; or (iii) “I guessed.” In our main analyses, the first option was considered “confident,” while the latter two were grouped together as “unconfident,” following a procedure from Smalle et al. (2022).

### Analyses

Only participants who completed the entire experiment were included in the analysis. Ten participants (spaced: 6, massed: 4) dropped out between the last exposure phase and the test phase. We filtered out exposure trials with less than two correct responses (spaced: 1.4%, massed: 1.7% of trials) or more than two responses (spaced: 1.7%, massed: 1.5% of trials) given at a point in the stream when no target was presented. If more than 20 trials (out of 72) were filtered out, the data of the participant was discarded entirely (three participants, all in the spaced group). Next, we removed targets that were not detected (spaced: 4.6%, massed 4.7% of targets). Lastly, we filtered out RTs that were more than 2.5 standard deviations away from the mean, separately for each participant (spaced: 3.1%, massed 3.3% of RTs). Test trials were removed when the response took longer than 5 seconds after onset of the response display (spaced: 3.7%, massed: 3.9% of trials).

Analyses of variance (ANOVAs), *t* tests, Pearson correlations, and Bayesian equivalents were performed using Jamovi 1.6.23 (Sahin & Aybek, 2019). Bayesian analyses were used to determine if nonsignificant results provide evidence for the null hypothesis. We report BF_01_ and BF_excl_, both expressing the strength of evidence in favor of the null hypothesis. We used the default Cauchy distribution (scale = 0.707) as the prior (Keysers et al., 2020; Morey & Rouder, 2011) for all analyses, such that the BF provides a good indication of the strength of evidence against (BF > 1) or for (BF < 1) the null hypothesis. The verbal labels used to describe the strength of the evidence are based on an established classification (Jeffreys, 1998). We used an appropriate alternative test when a violation of the assumption of normality (Mann–Whitney *U*) or homogeneity of variances (Welch’s) was detected. Data files and analysis scripts are available online (https://osf.io/wkj5c).

## Results

### Exposure phase: Preregistered analyses

**Fig. 2:**
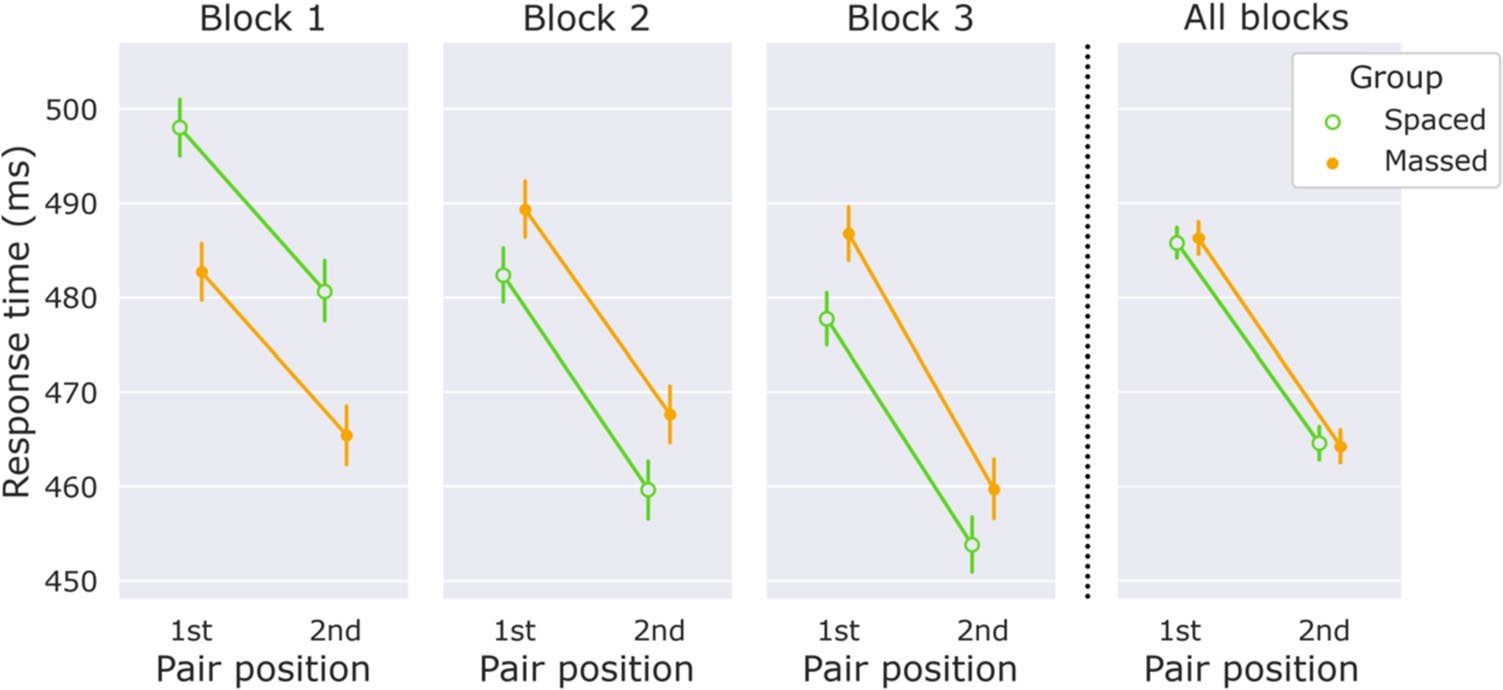
Response times in the exposure phase as a function of pair position (first/second) for the spaced and massed group separately. Error bars show 95% confidence intervals corrected for within-subject comparisons (Cousineau, 2005)

Figure 2 shows RTs in the exposure phase as a function of the syllable’s position within the pair (first/second), for the spaced versus the massed groups. We performed a repeated-measures ANOVA, with *RT* as the dependent variable, *pair position* (first/second) and *block* (1–3) as factors, and *group* (spaced/massed) as a between-subjects factor. Crucially, we found a reliable main effect of *pair position, F*(1,196) = 174.47, *p* < .001, η_p_^2^ = 0.47, indicating that participants used the learned syllable pairings to anticipate second-position targets. We also observed small but reliable effects of *block*, *F*(2,392) = 9.48, *p* < .001, η_p_^2^ = 0.05, *Block × Group*, *F*(2,392) = 11.14, *p* < .001, η_p_^2^ = 0.05, and *Block × Pair Position*, *F*(2,392) = 7.25, *p* < .001, η_p_^2^ = 0.04. All remaining effects were nonsignificant—namely, *group*, *F*(1,196) = 0.31, *p* = .579, BF_excl_ = 3.12, η_p_^2^ = 0, *Group × Pair Position, F*(1,196) = 0.18, *p* = .672, BF_excl_ = 9.63, η_p_^2^ = 0.00, and *Group × Pair Position × Block, F*(2,392) = 0.51, *p* = .599, BF_excl_ = 13.35, η_p_^2^ = 0.00. The BFs for the *Group × Pair Position* and *Group × Pair Position × Block* interactions can be taken as strong evidence that the spaced and massed groups showed equal RT benefits on the second pair position. Block-by-block comparisons between the groups (reported in the Supplementary Materials) confirm this.

Planned block-by-block comparisons revealed that the benefit of the second pair position was significant in every block for the spaced group, with all *p* values < .001 and *d* values > 0.68, as well as for the massed group, with all *p* values < .001 and *d* values > 0.58. We conclude that the RT benefit of the second over the first pair position was robust and occurred already in the first block. The results regarding the effect of *stream position,* referring to the position of the target among the four targets per stream, are reported in the Supplementary Materials.

### Exposure phase: Explorative analyses

To investigate the development of learning between blocks (irrespective of group), we performed a repeated-measures ANOVA, with the *SL index* as the dependent variable and *block* as the factor. We found a significant effect of *block*, *F*(2,197) = 7.27, *p* < .001, η_p_^2^ = 0.04. Post-hoc Bonferroni comparisons revealed an increase in the SL index between Blocks 1 and 3, *t*(197) = 3.54, *p =* .002, *d* = 0.25, but no significant difference between Blocks 1 and 2, *t*(197) = 2.26, *p =* .074, BF_01_ = 1.04, *d* = 0.16, or Blocks 2 and 3, *t*(197) = 1.66, *p =* .295, BF_01_ = 3.26, *d* = 0.12.

### Testing phase: Preregistered analyses

**Fig. 3:**
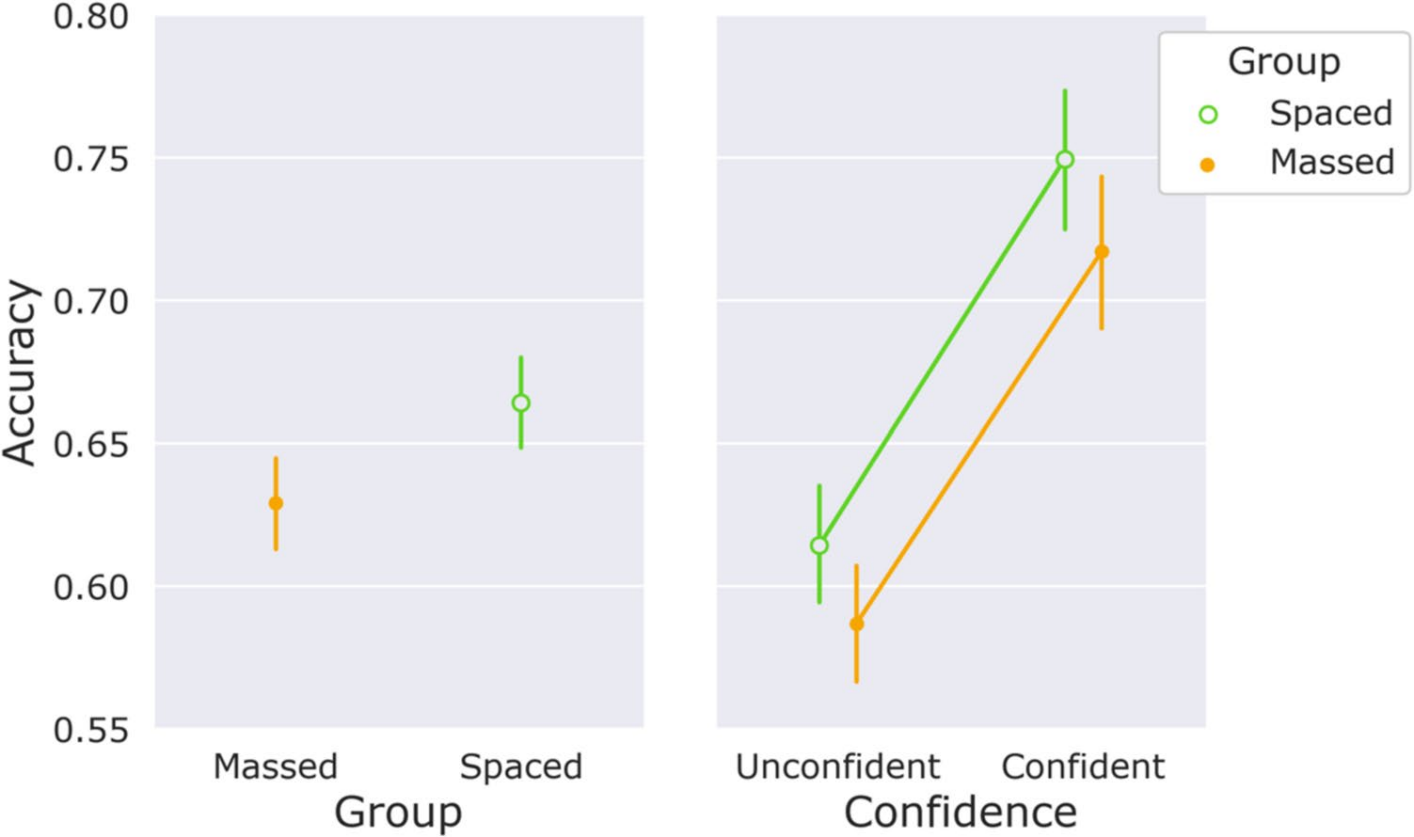
Accuracy in the testing phase as a function of group (left panel) and as a function of confidence (right panel) separated by group. Error bars show 95% confidence intervals

Figure 3 shows response accuracy in the testing phase for the spaced and the massed group. In the spaced group, accuracy was above chance level (0.5), *t*(98) = 54.3, *p <* .001, *d* = 5.45. This held true for confident responses (34.5% of the responses), *t*(94) = 35.7, *p <* .001, *d* = 3.66, as well as unconfident responses (65.5% of the responses), *t*(97) = 44.6, *p <* .001, *d* = 4.50. In the massed group accuracy was also above chance, *t*(98) = 66.7, *p <* .001, *d* = 6.7, for confident responses, *t*(90) = 32, *p <* .001, *d* = 3.35, as well as unconfident responses, *t*(97) = 49.2, *p <* .001, *d* = 4.97. This indicates that participants in both groups had explicit as well as implicit knowledge. We performed a repeated-measures ANOVA, with *confidence* as factor and *group* as a between-subjects factor, resulting in a main effect of *confidence*, *F*(1,182) = 69.18, *p* < .001, η_p_^2^ = 0.28, and nonsignificant effects of *group*, *F*(1,182) = 2.6, *p* = .108, η_p_^2^ = 0.01, BF_excl_ = 2.78, and *Group × Confidence, F*(1,182) = 0.03, *p* = .859, η_p_^2^ = 0.0, BF_excl_ = 4.76. The BF for *Group × Confidence* can be taken as evidence that the spacing benefit was equal for confident and unconfident responses. Notably, the main effect of *group* in the ANOVA was nonsignificant, but we lost power due to missing values for confident (16 participants) and unconfident (two participants) accuracies. Crucially, when we compared overall testing phase accuracy, we observed significantly higher accuracy in the spaced group compared with the massed group, Welch’s *t*(184) = 2.19, *p* = .030, *d* = 0.31. We have replicated these results in a mixed-effects logistic regression model (reported in the supplementary materials). While not preregistered, it could be argued that such a model is better suited for this type of data (Jaeger, 2008).

### Testing phase: Explorative analyses

To investigate whether participants had any awareness of the regularities, we first manually coded the answers to the open-ended Q1 (see Methods). We found that 59% (spaced: 61%, massed: 57%) of participants made mention of the word “pattern(s)” or gave a description thereof. However, when asked how many sounds were in the repeating subgroups (Q2), the median answer was 6 (*SD* = 4.4), and only 2.5% of participants (spaced: 0.5%, massed: 2%) gave the correct answer of 2. Some correlations regarding the self-reported wakefulness scores are reported in the Supplementary Materials.

## Discussion

In an auditory statistical learning paradigm, we observed a significant benefit of spaced over massed learning of novel embedded syllable patterns after a delay period. In the testing phase (2 weeks after the last exposure delay), the spaced group showed higher accuracy in identifying the learned pairs from the foils. Participants who had been exposed to the syllable pairings across 3 days outperformed those who received all exposure at once on a single day, even though both groups had the same total time of pattern exposure and learning during exposure (as measured by the RT benefit for targets that came second within a pair) was highly comparable for the spaced and massed groups. We interpret this as evidence for a spacing effect for auditory statistical learning. This suggests that the spacing effect, which is already well-established for many other forms of learning, also applies to auditory statistical learning.

While our findings were derived from a controlled experimental design, they highlight the importance of the timing of language learning both outside and inside the laboratory. Given the key role of auditory statistical learning for speech segmentation and natural language learning (Alexander et al., 2023; Pelucchi et al., 2009), our results further strengthen the call for incorporating spaced learning practices in language learning and education more broadly (Cull, 2000). On the other hand, they also have important implications for the research efforts on statistical learning. Whereas the majority of statistical learning in everyday life likely takes place over periods that span at least multiple days (akin to the spaced group in our design), exposure in statistical learning experiments typically takes no more than a few minutes (Frost et al., 2019), in a single session on a single day (for rare exceptions of multiday training, see Alexander et al., 2023; Chetail 2017). The finding that spaced learning benefits auditory statistical learning implies that such investigations are likely to underestimate participants’ statistical learning abilities.

Multiple mechanisms have been proposed to drive the spacing benefit, and it seems likely that a combination of forces simultaneously determine the effect (Gerbier & Toppino, 2015). According to the *deficient processing hypothesis*, a quickly repeated (i.e., massed) occurrence of a stimulus is processed less deeply (Johnston & Uhl, 1976; Magliero, 1983), resulting in decreased encoding quality. This more superficial encoding could reflect a conscious decrease in effort but has also been observed in incidental learning and implicit memory tasks (Greene, 1989, 1990). The decreased encoding quality for quickly repeated items has been linked to priming effects (Russo et al., 1998) and repetition suppression effects (Henson, 2003; Van Strien et al., 2007; Van Turennout et al., 2003) in behavioral and neuroimaging studies, respectively. Aside from encoding, in multiday spacing studies such as the present one, the spacing benefit likely also relies at least in part on the benefits of sleep for memory consolidation (Rasch & Born, 2013), which have also been observed for statistical learning (Durrant et al., 2011, 2016). How these different factors contribute to the overall spacing effect requires further investigation.

### Learning during exposure: Rapid yet steady

The online index of learning during exposure revealed that the RT benefit for the second-position targets was already robust in the first block, consistent with earlier work demonstrating that adult listeners gain sensitivity to the statistical structure in speech rapidly (Batterink, 2017). However, this benefit increased from the first block (comprising repetitions 1–32 for each pair) to the third block (comprising repetitions 65–96). This suggests that despite rapid initial learning, the learning or its consequence in terms of anticipating upcoming stimuli might not asymptote as quickly as has previously been suggested (Siegelman et al., 2018), and instead indicators of statistical learning could become particularly pronounced after numerous repetitions (Bogaerts et al., 2020).

### The role of awareness in the spacing effect

There was some indication of awareness of the regularities among a majority of participants (59%), although very few (3%) participants could indicate the length of the repeated patterns correctly. The absent *Group × Confidence* interaction (BF_excl_ = 4.76) suggests that the spacing benefit was equal for confident and unconfident responses, which could be taken as evidence for the irrelevance of awareness for the spacing effect. There is no clear consensus in the literature regarding the spacing benefit for implicit memory (Greene, 1990; Nakata & Elgort, 2021; Parkin et al., 1990; Whyte et al., 2022). More research is required to investigate the role of awareness in the spacing effect. This could for example be investigated using a statistical learning paradigm with or without explicit instructions (e.g., Batterink et al., 2015).

### Avenues for future research

We have shown evidence for the benefit of spaced exposure in statistical learning, but more research is needed to investigate the impact of spaced exposure under different circumstances. For example, in the present study, each pair of syllables was repeated 32 times within a block. It is possible that there is a trade-off between “taking time” and still having sufficient repetitions within a learning session to create a memory trace that will survive the delay until the next pattern occurrence, so that in case there are fewer repetitions within a block it may be more beneficial to lump together several blocks. Similarly, a different spacing, delay between exposure and test, and number of blocks could impact the spacing effect. Furthermore, our study involved adult participants, and it is well-documented that statistical learning abilities vary in complex ways across development (Krogh et al., 2013; Shufaniya & Arnon, 2018), as do the processes of memory retention (Gómez, 2017). Given that statistical learning tasks can be used as a simulation of language learning, they may be useful in investigating spacing at various stages of language development.

## Supplementary Information

Below is the link to the electronic supplementary material.

Supplementary file1 (DOCX 297 KB)

## Data Availability

Data files and materials are available online (https://osf.io/wkj5c).

## Declarations

### Open practices

Data files and materials are available online (https://osf.io/wkj5c).

Analyses and methods were preregistered (https://aspredicted.org/e4mm3.pdf).

## References

[CR1] Abreu, R., Postarnak, S., Vulchanov, V., Baggio, G., & Vulchanova, M. (2023). The association between statistical learning and language development during childhood: A scoping review. *Heliyon, 9*(8), Article e18693. 10.1016/j.heliyon.2023.e18693 PMC10405008 37554804

[CR2] Alexander, E., Van Hedger, S. C., & Batterink, L. J. (2023). Learning words without trying: Daily second language podcasts support word-form learning in adults. *Psychonomic Bulletin & Review,**30*(2), 751–762. 36175820 10.3758/s13423-022-02190-1

[CR3] Arciuli, J., & Simpson, I. C. (2012). Statistical learning is lasting and consistent over time. *Neuroscience Letters,**517*(2), 133–135. 22561650 10.1016/j.neulet.2012.04.045

[CR4] Aslin, R. N. (2017). Statistical learning: A powerful mechanism that operates by mere exposure. *Wiley Interdisciplinary Reviews: Cognitive Science,* 8(1/2), Article e1373. 10.1002/wcs.1373 PMC5182173 27906526

[CR5] Bahrick, H. P., Bahrick, L. E., Bahrick, A. S., & Bahrick, P. E. (1993). Maintenance of foreign language vocabulary and the spacing effect. *Psychological Science,**4*(5), 316–321.

[CR6] Batterink, L. J. (2017). Rapid statistical learning supporting word extraction from continuous speech. *Psychological Science,**28*(7), 921–928. 28493810 10.1177/0956797617698226 PMC5507727

[CR7] Batterink, L. J., & Paller, K. A. (2019). Statistical learning of speech regularities can occur outside the focus of attention. *Cortex,**115*, 56–71. 30771622 10.1016/j.cortex.2019.01.013 PMC6513683

[CR8] Batterink, L. J., Reber, P. J., Neville, H. J., & Paller, K. A. (2015). Implicit and explicit contributions to statistical learning. *Journal of Memory and Language,**83*, 62–78. 26034344 10.1016/j.jml.2015.04.004 PMC4448134

[CR9] Bird, S. (2011). Effects of distributed practice on the acquisition of second language English syntax—ERRATUM. *Applied Psycholinguistics,**32*(2), 435–452.

[CR10] Bogaerts, L., Richter, C. G., Landau, A. N., & Frost, R. (2020). Beta-band activity is a signature of statistical learning. *Journal of Neuroscience,**40*(39), 7523–7530. 32826312 10.1523/JNEUROSCI.0771-20.2020 PMC7511193

[CR11] Brysbaert, M. (2019). How many participants do we have to include in properly powered experiments? A tutorial of power analysis with reference tables. *Journal of Cognition, 2*(1), Article 16. 10.5334/joc.72 PMC6640316 31517234

[CR12] Carew, T. J., Pinsker, H. M., & Kandel, E. R. (1972). Long-term habituation of a defensive withdrawal reflex in Aplysia. *Science,**175*(4020), 451–454. 17731371 10.1126/science.175.4020.451

[CR13] Cepeda, N. J., Vul, E., Rohrer, D., Wixted, J. T., & Pashler, H. (2008). Spacing effects in learning: A temporal ridgeline of optimal retention. *Psychological Science,**19*(11), 1095–1102. 19076480 10.1111/j.1467-9280.2008.02209.x

[CR14] Chetail, F. (2017). What do we do with what we learn? Statistical learning of orthographic regularities impacts written word processing. *Cognition,**163*, 103–120. 28319684 10.1016/j.cognition.2017.02.015

[CR15] Conway, C. M., & Christiansen, M. H. (2001). Sequential learning in non-human primates. *Trends in Cognitive Sciences,**5*(12), 539–546. 11728912 10.1016/s1364-6613(00)01800-3

[CR16] Cornell, E. H. (1980). Distributed study facilitates infants’ delayed recognition memory. *Memory & Cognition,**8*(6), 539–542. 7219174 10.3758/bf03213773

[CR17] Cousineau, D. (2005). Confidence intervals in within-subject designs: A simpler solution to Loftus and Masson’s method. *Tutorials in Quantitative Methods for Psychology,**1*(1), 42–45.

[CR18] Cull, W. L. (2000). Untangling the benefits of multiple study opportunities and repeated testing for cued recall. *Applied Cognitive Psychology: The Official Journal of the Society for Applied Research in Memory and Cognition,**14*(3), 215–235.

[CR19] Deisig, N., Sandoz, J.-C., Giurfa, M., & Lachnit, J. (2007). The trial-spacing effect in olfactory patterning discriminations in honeybees. *Behavioural Brain Research,**176*(2), 314–322. 17113657 10.1016/j.bbr.2006.10.019

[CR20] Duncan, D., & Theeuwes, J. (2020). Statistical learning in the absence of explicit top-down attention.” *Cortex: A Journal Devoted to the Study of the Nervous System and Behavior, 131,* 54–65. 10.1016/j.cortex.2020.07.006 10.1016/j.cortex.2020.07.006 32801075

[CR21] Durrant, S. J., Cairney, S. A., & Lewis, P. A. (2016). Cross-modal transfer of statistical information benefits from sleep. *Cortex,**78*, 85–99. 27017231 10.1016/j.cortex.2016.02.011

[CR22] Durrant, S. J., Taylor, C., Cairney, S., & Lewis, P. A. (2011). Sleep-dependent consolidation of statistical learning. *Neuropsychologia,**49*(5), 1322–1331. 21335017 10.1016/j.neuropsychologia.2011.02.015

[CR23] Ebbinghaus, H. (1885). *Über Das Gedächtnis: Untersuchungen Zur Experimentellen Psychologie* [On memory: Investigations into experimental psychology]. Duncker & Humblot.

[CR24] Emberson, L. L., Conway, C. M., & Christiansen, M. H. (2011). Timing is everything: Changes in presentation rate have opposite effects on auditory and visual implicit statistical learning. *Quarterly Journal of Experimental Psychology,**64*(5), 1021–1040. 10.1080/17470218.2010.538972 21347988

[CR25] Erickson, L. C., & Thiessen, E. D. (2015). Statistical learning of language: Theory, validity, and predictions of a statistical learning account of language acquisition. *Developmental Review,**37*, 66–108.

[CR26] Foerde, K., Knowlton, B. J., & Poldrack, R. A. (2006). Modulation of competing memory systems by distraction. *Proceedings of the National Academy of Sciences,**103*(31), 11778–1183. 10.1073/pnas.0602659103 PMC1544246 16868087

[CR27] Frost, R., Armstrong, B. C., & Christiansen, M. H. (2019). Statistical learning research: A critical review and possible new directions. *Psychological Bulletin, 145*(12), Article 1128. 10.1037/bul0000210 31580089

[CR28] Gao, Y., & Theeuwes, J. (2022). Learning to suppress a location does not depend on knowing which location. *Attention, Perception, & Psychophysics,**84*(4), 1087–1097. 10.3758/s13414-021-02404-z PMC9076749 35194772

[CR29] Gerbier, E., & Toppino, T. C. (2015). The effect of distributed practice: Neuroscience, cognition, and education. *Trends in Neuroscience and Education,**4*(3), 49–59.

[CR30] Gómez, R. L. (2017). Do infants retain the statistics of a statistical learning experience? Insights from a developmental cognitive neuroscience perspective. *Philosophical Transactions of the Royal Society B: Biological Sciences, 372*(1711). 10.1098/rstb.2016.0054 10.1098/rstb.2016.0054 PMC5124079 27872372

[CR31] Greene, R. L. (1989). Spacing effects in memory: Evidence for a two-process account. *Journal of Experimental Psychology: Learning, Memory, and Cognition,**15*(3), 371–377.

[CR32] Greene, R. L. (1990). Spacing effects on implicit memory tests. *Journal of Experimental Psychology: Learning, Memory, and Cognition,**16*(6), 1004–1011.

[CR33] Hauser, M. D., Newport, E. L., & Aslin, R. N. (2001). Segmentation of the speech stream in a non-human primate: Statistical learning in cotton-top tamarins. *Cognition,**78*(3), B53–B64. 11124355 10.1016/s0010-0277(00)00132-3

[CR34] Henson, R. (2003). Neuroimaging studies of priming. *Progress in Neurobiology,**70*(1), 53–81. 12927334 10.1016/s0301-0082(03)00086-8

[CR35] Jaeger, T. F. (2008). Categorical data analysis: Away from ANOVAs (transformation or not) and towards logit mixed models. *Journal of Memory and Language,**59*(4), 434–446. 19884961 10.1016/j.jml.2007.11.007 PMC2613284

[CR36] Jeffreys, H. (1998). *The theory of probability*. Oxford University Press.

[CR37] Johnston, W. A., & Uhl, C. N. (1976). The contributions of encoding effort and variability to the spacing effect on free recall. *Journal of Experimental Psychology: Human Learning and Memory,**2*(2), 153–160.

[CR38] Keysers, C., Gazzola, V., & Wagenmakers, E.-J. (2020). Using Bayes factor hypothesis testing in neuroscience to establish evidence of absence. *Nature Neuroscience,**23*(7), 788–799. 32601411 10.1038/s41593-020-0660-4 PMC7610527

[CR39] Krogh, L., Vlach, H. A., & Johnson, S. P. (2013). Statistical learning across development: Flexible yet constrained. *Frontiers in Psychology, 3*, Article 598. 10.3389/fpsyg.2012.00598 PMC3576810 23430452

[CR40] Lange, K., Kühn, S., & Filevich, E. (2015). “Just Another Tool for Online Studies” (JATOS): An easy solution for setup and management of web servers supporting online studies.” *PLOS ONE, 10*(6), Article e0130834. 10.1371/journal.pone.0130834 10.1371/journal.pone.0130834 PMC4482716 26114751

[CR41] Magliero, A. (1983). Pupil dilations following pairs of identical and related to-be-remembered words. *Memory & Cognition,**11*, 609–615. 6669029 10.3758/bf03198285

[CR42] Mathôt, S., Schreij, D., & Theeuwes, J. (2012). OpenSesame: An open-source, graphical experiment builder for the social sciences. *Behavior Research Methods,**44*(2), 314–324. 22083660 10.3758/s13428-011-0168-7 PMC3356517

[CR43] McClelland, J. L., McNaughton, B. L., & O’Reilly, R. C. (1995). Why there are complementary learning systems in the hippocampus and neocortex: Insights from the successes and failures of connectionist models of learning and memory. *Psychological Review,**102*(3), 419–457. 7624455 10.1037/0033-295X.102.3.419

[CR44] Melton, A. W. (1967). Repetition and retrieval from memory. *Science (New York, NY), 158*(3800), Article 532. 10.1126/science.158.3800.532-b 17749105

[CR45] Morey, R. D., & Rouder, J. N. (2011). Bayes factor approaches for testing interval null hypotheses. *Psychological Methods,**16*(4), 406–419. 21787084 10.1037/a0024377

[CR46] Nakata, T., & Elgort, I. (2021). Effects of spacing on contextual vocabulary learning: Spacing facilitates the acquisition of explicit, but not tacit, vocabulary knowledge. *Second Language Research,**37*(2), 233–260.

[CR47] Ordin, M., & Polyanskaya, L. (2021). The role of metacognition in recognition of the content of statistical learning. *Psychonomic Bulletin & Review,**28*, 333–340. 32869190 10.3758/s13423-020-01800-0

[CR48] Pagán, A., & Nation, K. (2019). Learning words via reading: Contextual diversity, spacing, and retrieval effects in adults. *Cognitive Science, 43*(1), Article e12705. 10.1111/cogs.12705 30648796

[CR49] Palan, S., & Schitter, S. (2018). Prolific.Ac—A subject pool for online experiments. *Journal of Behavioral and Experimental Finance,**17*, 22–27.

[CR50] Parkin, A. J., Reid, T. K., & Russo, R. (1990). On the differential nature of implicit and explicit memory. *Memory & Cognition,**18*(5), 507–514. 2233263 10.3758/bf03198483

[CR51] Pelucchi, B., Hay, J. F., & Saffran, J. R. (2009). Statistical learning in a natural language by 8-month-old infants. *Child Development,**80*(3), 674–685. 19489896 10.1111/j.1467-8624.2009.01290.x PMC3883431

[CR52] Perruchet, P., & Vinter, A. (1998). PARSER: A model for word segmentation. *Journal of Memory and Language,**39*(2), 246–263.

[CR53] Rasch, B., & Born, J. (2013). About sleep’s role in memory. *Physiological Reviews,**93*(2), 681–766. 23589831 10.1152/physrev.00032.2012 PMC3768102

[CR54] Rawson, K. A., & Kintsch, W. (2005). Rereading effects depend on time of test. *Journal of Educational Psychology,**97*(1), 70–80.

[CR55] Ren, J., Wang, M., & Arciuli, J. (2023). A meta-analysis on the correlations between statistical learning, language, and reading outcomes. *Developmental Psychology,**59*(9), 1626–1644. 10.1037/dev0001577 37384516 10.1037/dev0001577

[CR56] Rogers, J. (2015). Learning second language syntax under massed and distributed conditions. *Tesol Quarterly,**49*(4), 857–866.

[CR57] Rogers, J. (2017). The spacing effect and its relevance to second language acquisition. *Applied Linguistics,**38*(6), 906–911.

[CR58] Romberg, A. R., & Saffran, J. R. (2010). Statistical Learning and Language Acquisition”. *Wiley Interdisciplinary Reviews: Cognitive Science,**1*(6), 906–14. 21666883 10.1002/wcs.78 PMC3112001

[CR59] Russo, R., Parkin, A. J., Taylor, S. R., & Wilks, J. (1998). Revising current two-process accounts of spacing effects in memory. *Journal of Experimental Psychology: Learning, Memory, and Cognition,**24*(1), 161–172. 10.1037/0278-7393.24.1.161 9438957 10.1037//0278-7393.24.1.161

[CR60] Saffran, J. R., Aslin, R. N., & Newport, E. L. (1996). Statistical learning by 8-month-old infants. *Science,**274*(5294), 1926–1928. 8943209 10.1126/science.274.5294.1926

[CR61] Saffran, J. R., Newport, E. L., Aslin, R. N., Tunick, R. A., & Barrueco, S. (1997). Incidental language learning: Listening (and learning) out of the corner of your ear. *Psychological Science,**8*(2), 101–105.

[CR62] Sahin, M. D., & Aybek, E. C. (2019). Jamovi: An easy to use statistical software for the social scientists. *International Journal of Assessment Tools in Education,**6*(4), 670–692.

[CR63] Schapiro, A. C., Turk-Browne, N. B., Botvinick, M. M., & Norman, K. A. (2017). Complementary learning systems within the hippocampus: A neural network modelling approach to reconciling episodic memory with statistical learning. *Philosophical Transactions of the Royal Society B: Biological Sciences, 372*(1711), Article 20160049. 10.1098/rstb.2016.0049 PMC5124075 27872368

[CR64] Sherman, B. E., Graves, K. N., Huberdeau, D. M., Quraishi, I. H., Damisah, E. C., & Turk-Browne, N. B. (2022). Temporal dynamics of competition between statistical learning and episodic memory in intracranial recordings of human visual cortex. *Journal of Neuroscience,**42*(48), 9053–9068. 36344264 10.1523/JNEUROSCI.0708-22.2022 PMC9732826

[CR65] Sherman, B. E., & Turk-Browne, N. B. (2020). Statistical prediction of the future impairs episodic encoding of the present. *Proceedings of the National Academy of Sciences,**117*(37), 22760–2270. 10.1073/pnas.2013291117 PMC7502714 32859755

[CR66] Shufaniya, A., & Arnon, I. (2018). Statistical learning is not age-invariant during childhood: Performance improves with age across modality. *Cognitive Science,**42*(8), 3100–3115. 30276848 10.1111/cogs.12692

[CR67] Siegelman, N., Bogaerts, L., Christiansen, M. H., & Frost, R. (2017). Towards a theory of individual differences in statistical learning. *Philosophical Transactions of the Royal Society B: Biological Sciences, 372*(1711), Article 20160059. 10.1098/rstb.2016.0059 PMC5124084 27872377

[CR68] Siegelman, N., Bogaerts, L., Kronenfeld, O., & Frost, R. (2018). Redefining ‘learning’ in statistical learning: What does an online measure reveal about the assimilation of visual regularities? *Cognitive Science,**42*, 692–727. 28986971 10.1111/cogs.12556 PMC5889756

[CR69] Smalle, E. H. M., Daikoku, T., Szmalec, A., Duyck, W., & Möttönen, R. (2022). Unlocking adults’ implicit statistical learning by cognitive depletion. *Proceedings of the National Academy of Sciences, 119*(2), Article e2026011119. 10.1073/pnas.2026011119 PMC8764693 34983868

[CR70] Smalle, E. H. M., Muylle, M., Duyck, W., & Szmalec, A. (2021). Less is more: Depleting cognitive resources enhances language learning abilities in adults. *Journal of Experimental Psychology: General, 150*(12), Article 2423. 10.1037/xge0001058 33856850

[CR71] Theeuwes, J., Bogaerts, L., & van Moorselaar, D. (2022). What to expect where and when: How statistical learning drives visual selection. *Trends in Cognitive Sciences,**26*(10), 860–872. 10.1016/j.tics.2022.06.001 35840476 10.1016/j.tics.2022.06.001

[CR72] Thiessen, E. D. (2017). What’s statistical about learning? Insights from modelling statistical learning as a set of memory processes. *Philosophical Transactions of the Royal Society B: Biological Sciences, 372*(1711), Article 20160056. 10.1098/rstb.2016.0056 PMC5124081 27872374

[CR73] Toppino, T. C., & DiGeorge, W. (1984). The spacing effect in free recall emerges with development. *Memory & Cognition,**12*(2), 118–122. 6727633 10.3758/bf03198425

[CR74] Toro, J. M., Sinnett, S., & Soto-Faraco, S. (2005). Speech segmentation by statistical learning depends on attention. *Cognition,**97*(2), B25–B34. 16226557 10.1016/j.cognition.2005.01.006

[CR75] Toro, J. M., & Trobalón, J. B. (2005). Statistical computations over a speech stream in a rodent. *Perception & Psychophysics,**67*(5), 867–875. 16334058 10.3758/bf03193539

[CR76] Tully, T., Preat, T., Boynton, S. C., & Del Vecchio, M. (1994). Genetic dissection of consolidated memory in drosophila. *Cell,**79*(1), 35–47. 7923375 10.1016/0092-8674(94)90398-0

[CR77] Turk-Browne, N. B., Jungé, J. A., & Scholl, B. J. (2005). The automaticity of visual statistical learning. *Journal of Experimental Psychology: General,**134*(4), 552–564. 10.1037/0096-3445.134.4.552 16316291 10.1037/0096-3445.134.4.552

[CR78] Vadillo, M. A., Konstantinidis, E., & Shanks, D. R. (2016). Underpowered samples, false negatives, and unconscious learning. *Psychonomic Bulletin & Review,**23*(1), 87–102. 26122896 10.3758/s13423-015-0892-6 PMC4742512

[CR79] Vadillo, M. A., Linssen, D., Orgaz, C., Parsons, S., & Shanks, D. R. (2020). Unconscious or underpowered? Probabilistic cuing of visual attention. *Journal of Experimental Psychology: General,**149*(1), 160–181. 10.1037/xge0000632 31246061 10.1037/xge0000632

[CR80] Van Strien, J. W., Verkoeijen, P. P. J. L., Van der Meer, N., & Franken, I. H. A. (2007). Electrophysiological correlates of word repetition spacing: ERP and induced band power old/new effects with massed and spaced repetitions. *International Journal of Psychophysiology,**66*(3), 205–214. 17688964 10.1016/j.ijpsycho.2007.07.003

[CR81] Van Turennout, M., Bielamowicz, L., & Martin, F. (2003). Modulation of neural activity during object naming: Effects of time and practice. *Cerebral Cortex,**13*(4), 381–391. 12631567 10.1093/cercor/13.4.381

[CR82] Wegener, S., Wang, H.-C., Beyersmann, E., Reichle, E. D., Nation, K., & Castles, A. (2022). The effect of spacing versus massing on orthographic learning. *Reading Research Quarterly,**58*(3), 361–372.

[CR83] Whyte, S., Edmonds, A., Palasis, K., & Gerbier, E. (2022). *ESPACE L2: Exploring spacing effects in explicit and implicit online learning of L2 English.* Paper presented at the EUROCALL 2022 Conference, Reykjavik, Iceland.

[CR84] Wiseheart, M., Küpper-Tetzel, C. E., Weston, T., Kim, A. S. N., Kapler, I. V., & Foot-Seymour, V. (2019). Enhancing the quality of student learning using distributed practice. In J. Dunlosky & K. A. Rawson (Eds.), *The Cambridge handbook of cognition and education* (pp. 550–583). Cambridge University Press.

