# Supplementary material for: Taking time: Auditory statistical learning benefits from distributed exposure
Source: Psychon Bull Rev. 2025 Jan 17;32(4):1562–71. doi: 10.3758/s13423-024-02634-w (PMC12325555; doi:10.3758/s13423-024-02634-w)
Supplement: Supplementary file 1 — Supplementary file1 (DOCX 297 KB) [file 13423_2024_2634_MOESM1_ESM.docx]

**Supplementary Materials**

*Exposure phase: preregistered analyses per block*

We took the RT benefit of the second pair position (RT_pair position 1_ − RT_pair position 2_) as an online index of statistical learning (SL index). Mirroring the lack of a *group × pair position* interaction (results, first paragraph)*,* we found no difference in SL index between the spaced and massed group, *U*(196) = 4801, *p =* .805, *BF_01_* = 5.88, *d* = 0.05. When we did planned comparisons per block, we found no group difference in any of the blocks, with all *p*’s > .361, *BF_01_*’s > 4.35 and *d*’s < 0.13. Since a *BF_01_* > 3 can be taken as substantial evidence for the absence of a difference, we conclude that the spaced and massed group showed comparable learning during the exposure phase.

*Exposure phase: preregistered analyses on stream position*

Figure S1 shows response times as a function of pair position for each of the stream positions, meaning the position of the target among the four targets per stream. We took the SL index and used it to investigate the effect of *stream position.* If the pairs were learned robustly, the SL index should already be significant for the first target in a stream (i.e. stream position 1), and a gain in the learning index for increasing stream positions reflects additional learning within the trial.


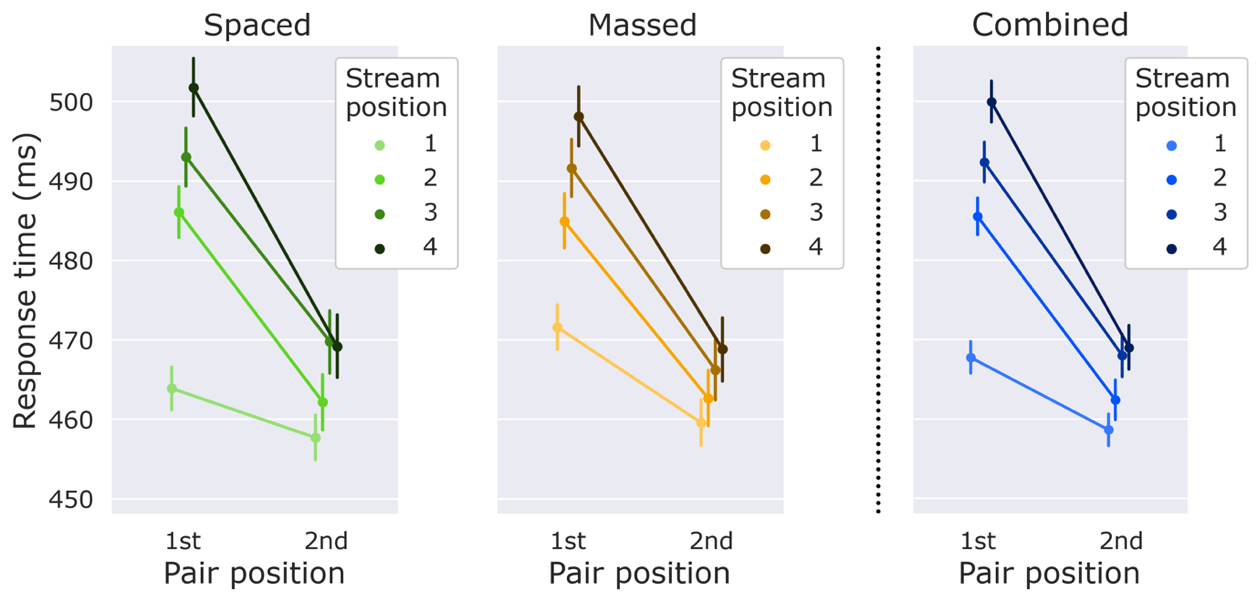


Figure S1. Response times in the exposure phase as a function of pair position (first/second), separated by stream position. 'Stream position' refers to the position of the target among the four targets per stream. Error bars show 95% confidence intervals corrected for within-subject comparisons (Cousineau 2005).

We performed a repeated-measures ANOVA with the *SL index* as the dependent variable, *stream position* as factor, and *group* as between-subject factor. We found a significant effect of *stream position*, *F*(3,588) = 28.22, *p* < .001, *η^2^_p_* = 0.13, and non-significant effects for *group*, *F*(1,196) = 0.11, *p* = .737, *BF_excl_* = 9.12, *η^2^_p_* = 0.00, and *group × stream position*, *F*(3,588) = 1.35, *p* = .259, *BF_excl_* = 30.21, *η^2^_p_* = 0.01. Post-hoc Bonferroni comparisons for *stream position* revealed no difference between stream position 2 and 3, *t*(197) = 0.52, *p* = 0.603, *d* = 0.04, *BF_01_* = 11.01, and significant differences between all other stream positions, with all *p*’s < .029 and *d*’s > 0.20.

Planned comparisons showed that the SL index was significant across all stream positions for the spaced group, with all *p*’s < .003 and *d*’s > 0.31, as well as for the massed group, with all *p*’s < .001 and *d*’s > 0.52. We conclude that there is evidence for learning already for the first target in a stream.

Planned comparisons comparing the learning score in both groups per stream position reveal no group difference for any of the stream positions, with all *p*’s > .063, *BF_01_*’s > 1.28 (all *BF_01_*’s > 5.56, except for pair position 1) and *d*’s < 0.27.

*Exposure phase: RT distributions*

In our results, we have used a 100-1200 ms time window after target onset to define a response as correct. Figure S3 shows the RT distributions given that time window (left) and an alternative time window of -300 to 1200 ms (right).


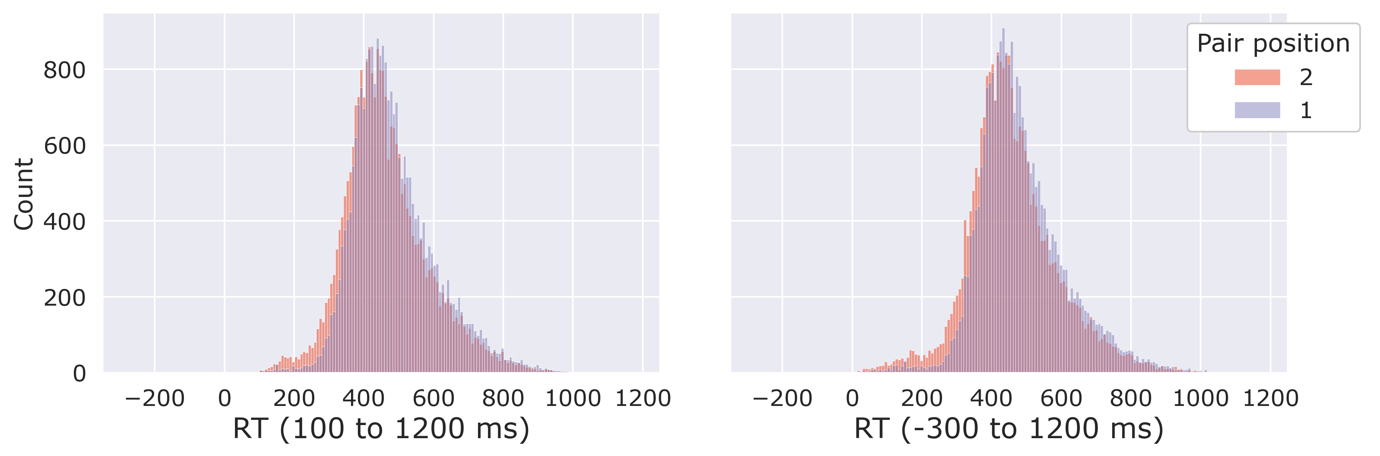


*Figure S3. Response time distributions for two different time windows, separated by pair position.*

Below we repeat the main RT analyses using the time window of -300 to 1200 to identify a response as a hit. We performed a repeated-measures ANOVA with *RT* as the dependent variable, *pair position* (first/second) and *block* (1-3) as factors, and *group* (spaced/massed) as between-subject factor. We found a reliable main effect of *pair position,* *F*(1,196) = 186.58, *p* < .001, *η^2^_p_* = 0.49, indicating that participants used the learned syllable pairings to anticipate second-position targets. We also observed small but reliable effects of *block*, *F*(2,392) = 10.99, *p* < .001, *η^2^_p_* = 0.05, *block × group*, *F*(2,392) = 12.82, *p* < .001, *η^2^_p_* = 0.06, and *block × pair position*, *F*(2,392) = 6.42, *p* = .002, *η^2^_p_* = 0.03. All remaining effects were non-significant, namely *group*, *F*(1,196) = 0.32, *p* = .573, BF_excl_ = 2.83, *η^2^_p_* = 0.00, *group × pair position, F*(1,196) = 0.03, *p* = .851, BF_excl_ = 8.88, *η^2^_p_* = 0.00, and *group × pair position × block, F*(2,392) = 0.48, *p* = .617, BF_excl_ = 21.05, *η^2^_p_* = 0.00.

*Testing phase: explorative correlations*

The SL index from the exposure phase was correlated with the accuracy during testing, *r* = 0.45, *p* < .001, indicating their validity as measures of statistical learning. Average wakefulness during exposure did not correlate with SL index, *r* = 0.02, *p* = .766, *BF_01_* = 10.70, or with test phase accuracy, *r* = 0.05, *p* = .498, *BF_01_* = 8.95. Similarly, average wakefulness during testing did not correlate with test phase accuracy, *r* = −0.10, *p* = .146, *BF_01_* = 3.94.

*Testing phase: mixed-effects logistic regression*

We ran a mixed-effects logistic regression with *correct response* (0/1) as the dependent variable, *confidence response* (recalled/vaguely familiar/guessed), *group* (spaced/massed), and their interaction as factors, and *subject* as the cluster variable. Both factors were deviation coded. Using the maximal effects structure justified by the design that allowed the model to converge (Barr et al. 2013), we included a by-subject intercept as random effect. The analysis was performed with Jamovi 2.3.28 (R Core Team 2013; Sahin and Aybek 2019) using the GAMLj module (Gallucci 2019) and the bobbyqa optimizer. The model included 6860 observations and produced an AIC of 8748. The factor *Group* produced a Beta estimate of 0.076 (SE = 0.037). Omnibus test results are shown in Table 1 below.

| Table 1. Fixed Effect Omnibus tests | | | | | | | |
| --- | --- | --- | --- | --- | --- | --- | --- |
|  | | **X²** | | **df** | | **p** | |
| Group |  | 4.122 |  | 1.00 |  | 0.042 |  |
| Confidence response |  | 120.825 |  | 2.00 |  | < .001 |  |
| Group ✻ confidence response |  | 0.534 |  | 2.00 |  | 0.766 |  |
|  | | | | | | | |

**References**

Barr, Dale J., Roger Levy, Christoph Scheepers, and Harry J. Tily. 2013. “Random Effects Structure for Confirmatory Hypothesis Testing: Keep It Maximal.” *Journal of Memory and Language* 68(3):255–78.

Cousineau, Denis. 2005. “Confidence Intervals in Within-Subject Designs: A Simpler Solution to Loftus and Masson’s Method.” *Tutorials in Quantitative Methods for Psychology* 1(1):42–45.

Gallucci, M. 2019. “GAMLj: General Analyses for Linear Models. [Jamovi Module]. 2019.” *URL: Https://Gamlj. Github. Io*.

R Core Team. 2013. “R: A Language and Environment for Statistical Computing.”

Sahin, Murat Dogan, and Eren Can Aybek. 2019. “Jamovi: An Easy to Use Statistical Software for the Social Scientists.” *International Journal of Assessment Tools in Education* 6(4):670–92.
